# Malignant Transformation of Oral Lichen Planus: A Case Report

**DOI:** 10.7759/cureus.38432

**Published:** 2023-05-02

**Authors:** Soukaïna Oujdad, Ihsane Ben Yahya

**Affiliations:** 1 Department of Oral Medicine and Oral Surgery, Faculty of Dental Medicine of Casablanca, Casablanca, MAR

**Keywords:** oral mucosal lesions, potentially malignant disorder, malignant transformation, squamous cell carcinoma, oral lichen planus

## Abstract

Oral lichen planus (OLP) is a chronic auto-immune disease associated with cell-mediated immunologic dysfunction whose etiology remains unknown. Malignant transformation into squamous cell carcinoma (SCC) is the most feared complication of OLP. It is actually recognized as a potentially malignant disorder with an unspecified risk of transformation, and it is suggested to closely monitor diagnosed patients.

In this paper, we discuss the case of a 62-year-old female patient, a non-smoker, and non-drinker, who initially presented with painful erosive lesions on the edentulous mandibular ridge, the buccal mucosa, and the floor of the mouth. Clinical and histological findings led to the diagnosis of OLP with no signs of oral epithelial dysplasia. Malignant transformation into SCC was reported eight months after the first presentation.

## Introduction

Oral lichen planus (OLP) is a chronic inflammatory disorder of unknown etiology with characteristic relapses and remissions, displaying white reticular lesions accompanied or not by atrophic, erosive, ulcerative, and/or plaque-type areas. Lesions are frequently bilaterally symmetrical [1]. Malignant transformation of OLP has been reported in isolated and longitudinal studies [2,3].

## Case presentation

A 62-year-old female patient presented to our department with multiple oral ulcerations and persistent burning sensations that had developed four weeks before the first consultation. The patient reported that her symptoms did not respond to local antiseptic treatments prescribed by her general practitioner. The patient was a non-smoker and did not consume alcohol. She was diagnosed with rheumatoid arthritis four years prior and was, at the time of the consultation, on hydroxychloroquine (Plaquenil®).

**Figure 1:**
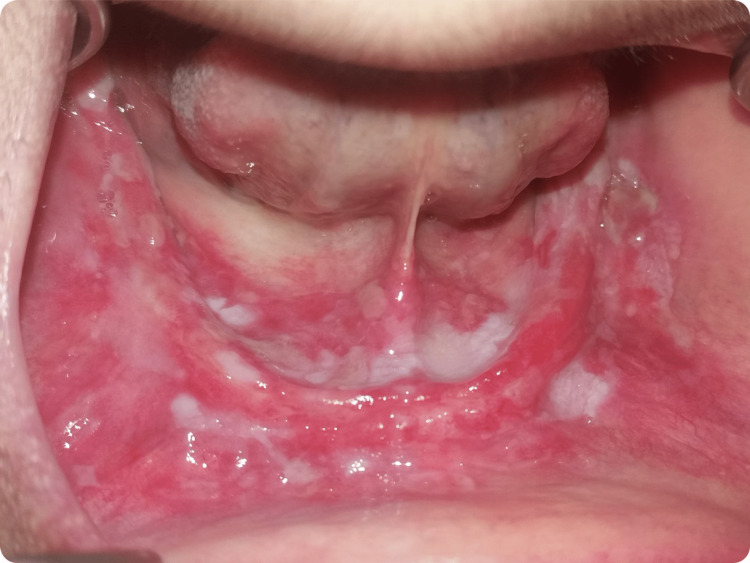
Intraoral examination at the time of the first consultation Combined lesions were noted on the edentulous mandibular ridge, the floor of the mouth, and the buccal mucosa. An inflammatory base covered by ulcerations and a whitish pseudomembrane. No induration was noted.

A physical examination revealed a healthy-appearing and responsive woman. No facial asymmetry or lymph node enlargement were noted. Intraoral examination revealed bilateral erosive lesions on the edentulous mandibular ridge, the buccal mucosa, and the floor of the mouth. The lesions presented as an inflammatory base with scalloped borders, covered with multiple ulcerations partially coated with pseudomembrane. The lesions were extremely sensitive to the touch, and no induration was noted on palpation. The maxillary arch was edentulous and did not present any lesions (Figure 1).

**Figure 2:**
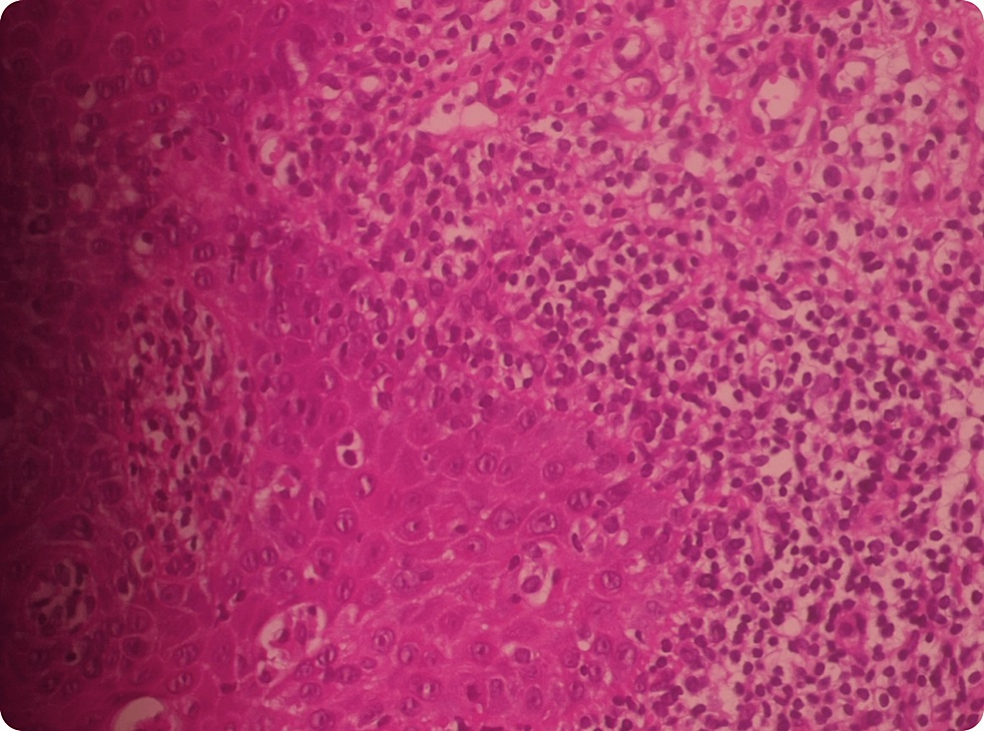
Histological features indicating oral lichen planus Histological examination revealed the presence of epithelial hyperkeratosis and a dense band of lymphocytic infiltrate in the superficial lamina. The basal layer showed liquefaction degeneration.

Complete blood count (CBC), erythrocyte sedimentation rate (ESR), hepatitis B and C, and human immunodeficiency virus (HIV) serology testing did not reveal any particularities. A first biopsy was performed under local anesthesia, indicating the presence of epithelial hyperkeratosis and a dense band of lymphocytic infiltrate in the superficial lamina. The basal layer showed liquefaction degeneration. These findings concluded with an erosive oral lichen planus with no signs of epithelial dysplasia (Figure 2). The patient received degressive systemic corticosteroid therapy for five weeks: 60mg of prednisolone for seven days, followed by 40 mg for seven days, 20mg for seven days, then 10 mg for seven days, followed by local corticosteroid therapy based on betamethasone. Subsequently, she showed up regularly to her check-up appointments, every 15 days, and manifested a significant decrease in clinical symptoms.

**Figure 3:**
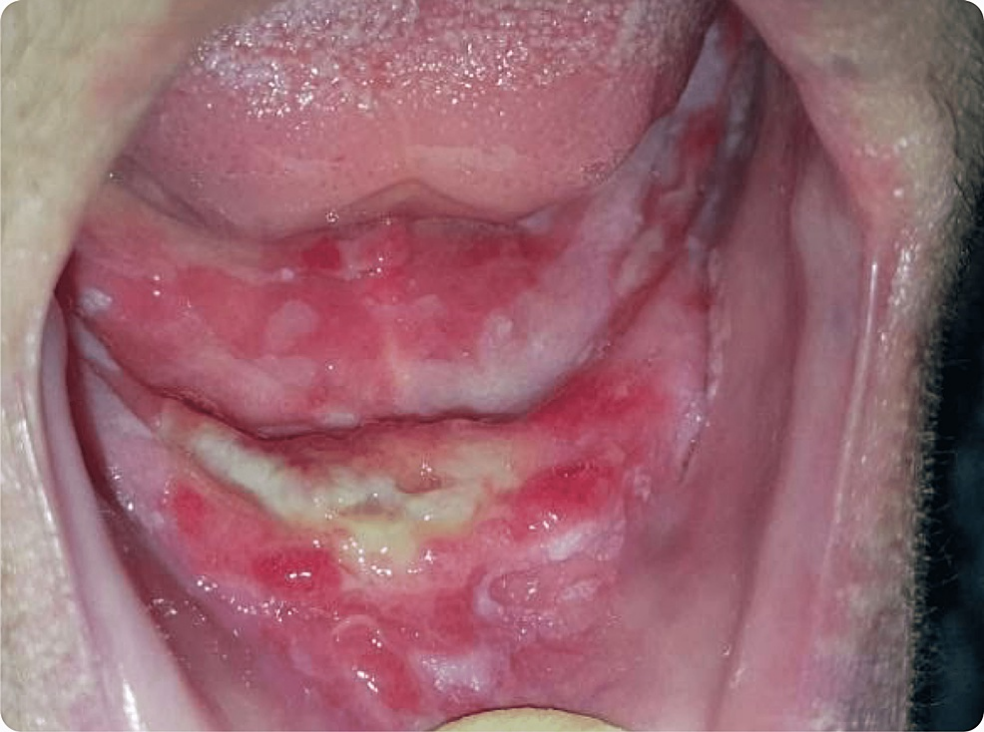
Intraoral examination, eight months later Necrotic ulceration with irregular borders on the anterior mandibular ridge, accompanied by intense pain on palpation.

**Figure 4:**
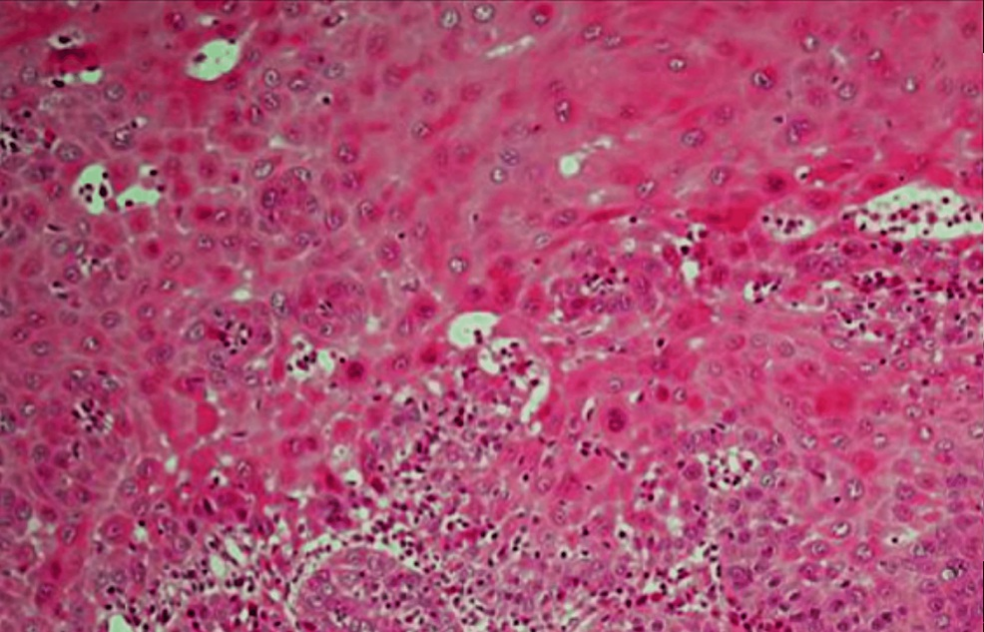
Histological examination showed features of well-differentiated squamous cell carcinoma Histological examination showed architectural disorganization, ulcerated infiltrating carcinomatous proliferation, cohesive cell clusters, and a loss of limit between the epithelium and the lamina propria.

Eight months later, the patient presented with extreme burning sensations. Oral examination revealed yellowish necrotic ulcerations with ill-delineated and slightly elevated borders. No induration was noted (Figure 3). A second biopsy was scheduled, which revealed the presence of a tumorous squamous mucosa occupied by ulcerated infiltrating carcinomatous proliferation and cohesive cell clusters, indicating a well-differentiated squamous cell carcinoma (SCC) (Figure 4).

The chest X-ray showed no particularities. A cervicofacial CT scan revealed the presence of two irregular nodular thickenings of the mandibular gingiva infiltrating the underlying tissues and two small bilateral jugulo-carotid lymphadenopathies. No bone lysis was noted.

The oncologist’s conclusion was that stage T1N1M0 was infiltrating SCC. The treatment consisted of a conservative mucosal and bone curettage, associated with radical neck dissection, under general anesthesia. Surgical margins and sampled lymph nodes were negative for SCC.

## Discussion

The first evidence of malignant OLP transformation was published in 1910 by Hallopeau et al. [4]. Since then, multiple cases have been reported [5]. Currently, the WHO recognizes OLP as a potentially malignant disorder with an unspecified risk of transformation and suggests closely monitoring diagnosed patients [1].

In recent studies, the malignant transformation rate of OLP ranged from zero to 3.5%. The average time from diagnosis of OLP to malignant transformation ranged from six to 156 months [6-8]. In our case, the malignant transformation occurred relatively early (eight months) after the diagnosis of OLP. In Fitzpatrick SG et al.'s report, the patients' average age at the onset of SCC, after the diagnosis of OLP, was 60.8 years. A slight predominance of female patients was also reported [7].

In most reports, the rate of erosive and atrophic types was higher than in other forms, which is in accordance with our case [5]. Aghbari S.A. et al. found a higher incidence of malignant transformation of OLP among smokers, alcohol consumers, and HCV-infected patients [6]. Although the evidence of tobacco smoking has been widely established as a carcinogen, the relationship between tobacco smoking and the malignant transformation of OLP is debatable. Other risk factors were suggested by Giuliani M. et al., including the erosive type, the female gender, and the tongue site [7]. In our report, we believe that severe chronic inflammation is the main reason for malignant transformation.

The mechanism by which OLP transforms into SCC is not entirely understood. The latest findings speculate that OLP has a tumor-like microenvironment that contributes to its malignant transformation. Cellular signals and mediators of inflammation (such as interleukin 4 (IL4) and IL-6), implicated in chronic inflammation, play critical roles in increasing the sensitivity of oral keratinocytes to exogenous mutagens [9].

Surgical resection is the common treatment for SCC. The clinical stage at presentation is an important predictor of survival. Significant histopathologic predictors of outcome include the depth of invasion of the primary tumor, positive margins of surgical resection, perineural invasion, and major extracapsular nodal extension [10]. In our case, the diagnosis of SCC was relatively early. The absence of bone extension allowed the patient to avoid resection surgeries. The CT scan revealed the presence of two bilateral lymphadenopathies. In accordance with the patient’s age, the presence of comorbidity (rheumatoid arthritis), and the presence of lesions on the floor of the mouth, radical neck dissection was the treatment of choice. To enhance healing, a topical application of 0.2% hyaluronic acid gel was prescribed for three months after the surgery.

## Conclusions

It is evident that OLP does carry a non-negligible risk of malignant transformation. Patient education and thorough clinical follow-ups are keys to early diagnosis. Dentists and oral surgeons should not hesitate to carry out a biopsy every time a structural change is noted.
